# Dietary Intake of 14–15-Year-Old Faroese Adolescents by an Online Assessment Tool and Associations with Wellbeing and Health Behaviour

**DOI:** 10.3390/nu16162621

**Published:** 2024-08-09

**Authors:** Unn Thomsen, Anna Sofía Veyhe, Marin Strøm

**Affiliations:** 1 Faculty of Health Sciences, University of the Faroe Islands, FO-100 Tórshavn, Faroe Islands; unnthomsen@gmail.com (U.T.); annasv@setur.fo (A.S.V.); 2 Department of Epidemiology Research, Statens Serum Institut, 2300 Copenhagen, Denmark

**Keywords:** adolescent dietary assessment, 24 h dietary recall, wellbeing and health, foods and nutrients

## Abstract

There is robust evidence linking diet and physical activity to major public health concerns such as cardiovascular diseases, type 2 diabetes, and osteoporosis. Dietary habits of children and adolescents are frequently discussed in health policy debates due to their modifiability, making them viable targets for prevention and health promotion initiatives. This study aimed to assess the dietary habits of 14–15-year-old Faroese adolescents using an online 24 h recall tool, examining their intake relative to public recommendations and exploring associations with health behaviour and wellbeing. A total of 78 participants (45 girls, 33 boys), with a mean age of 14.3 years, recorded their food intake and completed a questionnaire. Results indicated a higher intake of saturated fats and sugar and a lower intake of dietary fibre, *n*-3 fatty acids, fruits, and vegetables compared to recommendations. Healthier food intake was associated with better health behaviour and wellbeing. This very first study of Faroese adolescents’ overall diet underscores the need for health-promoting interventions, and suggests the feasibility of using an online 24 h recall tool for dietary assessment in this age group, albeit with necessary adjustments for Faroese language and traditional foods.

## 1. Introduction

There is thorough evidence that diet impacts people’s health and quality of life. According to the ‘European Food and Nutrition Action Plan 2015–2020’ from the WHO, an unhealthy diet can lead to malnutrition, obesity, autoimmune diseases, Parkinson’s, cardiovascular diseases, cancer, and type 2 diabetes, among others. In contrast, a healthy diet can prevent the same diseases [1]. An unhealthy diet has significant social and economic costs for individuals and society and is a modifiable factor [2].

The Faroe Islands are an archipelago in the North Atlantic and a self-governing part of the Kingdom of Denmark with a population of approx. 54,000, where the fertility rate and life expectancy are among the highest in the world [3]. Traditionally, marine animals have constituted a major part of the diet in the Faroe Islands [4]. Pilot whale meat and blubber have been part of the Faroese diet for centuries, but due to high levels of contaminants, a recommendation was issued in 2008 that these are no longer considered fit for human consumption [5]. However, even though fishery is still the nation’s primary leading industry [6], in recent years, the diet seems to have shifted towards a more Western-type diet, with higher intakes of sugar and saturated fat and less seafood. There is little evidence regarding the dietary intake of the Faroese population, but the general diet was assessed in three studies at three time points: in 1981–1982 (*n* = 331) [7], 2000–2001 (*n* = 148) [8], and 2013–2016 (*n* = 703) [9]. These studies have documented a shift in diet from traditional food sources to a more Western-style diet, and in particular, a marked drop in intake of fish and marine animals [9].

To the best of our knowledge, there is no data available on dietary intake for Faroese adolescents, and young people generally seem to constitute an overlooked group in research on dietary patterns and behaviours [10]. However, there is evidence that young people tend to adopt more unhealthy diets with a high content of fat, sugar, and fast food and few vegetables [11]. Still, studies are lacking that examine the causes for this worsening of adolescent nutritional habits [12]. Being in the transition phase from childhood to adulthood, moving towards more independence has a significant impact on both their current and future health. Managing this increasing autonomy can imply health behaviours tracking into adulthood and thus affecting future health [11]. Therefore, a healthy diet is particularly important during adolescence to ensure the intake of micronutrients necessary for optimal physical growth and development, cognitive performance, and mental health [13,14,15]. Diet may not affect young people’s current health to a large extent. However, unhealthy dietary patterns are shown to affect their metabolism and the relationship between body fat and muscles in a negative way [16]. Furthermore, in addition to the long-term effects of so-called lifestyle diseases, diet is also closely related to wellbeing and quality of life, both in the short and long term [17,18].

Therefore, we conducted a small exploratory study to assess Faroese adolescents’ general diet using a web-based 24 h recall tool [19]. The aim was to describe the dietary intake, to evaluate the extent to which the Faroese adolescents adhere to relevant recommendations, and to explore associations with measures of wellbeing and health behaviour.

## 2. Materials and Methods

Four schools were approached to enable data collection during school hours, aiming at different geographic and socioeconomic areas of the Faroes (central, west, north/east, and south). Once approved by the school principal, the primary teacher of each 8th grade was approached as gatekeeper and asked to send out written information provided by the project responsible to parents of all pupils in 8th grade in the respective schools. The parents provided consent on behalf of their child, and only those adolescents who brought the signed consent form to school on the day of data collection could participate. The national data authority approved the project.

Data collection consisted of online dietary recording; a paper questionnaire asking about gender, age, self-rated health, leisure time activities, screen time, sleep patterns, and frequency of headache and stomach ache; and weight and length measurements. During the data collection, the children were asked to go into a different room one by one to measure weight and length.

For the online 24 h recall, each child was given a URL code to log into an online registration form from myfood24 (Leeds, UK) [20], where they had to enter details of their food intake throughout the previous day, from waking up to going to bed. The online tool is in Danish, the second language for most Faroese that is taught in school from the 3rd grade, so the project responsible for the data collection initially instructed the whole group, including information on some habitual foods that are named quite differently in Faroese and Danish.

The Danish database used for the calculation of macro- and micronutrients by the Danish version of myfood24 was built by the Danish Cancer Society Research Center in 2017 [21]. It includes 1668 items covering adult and infant foods and is described in detail elsewhere [21]. In short, it consists of all edible items from the Danish Food Composition Table ‘FRIDA Food’ [22], complemented with values for cooked varieties of all raw items from food composition tables from Sweden and England and the branded myfood24 database [23]. Images of various portion sizes obtained from the Danish Food Institute were incorporated. Within the online tool myfood24, each food item was multiplied by the specified portion size and linked to its corresponding food composition table to directly calculate food, energy, and nutrient intake in myfood24 [21].

Data on intake of macro- and micronutrients in amount/day and daily frequency of fruit and vegetable consumption from myfood24 was imported to the statistical program SPSS. The paper questionnaires and weight and length measurements were manually entered into the same database. A statistical investigation of outliers was performed but did not result in the exclusion of any observations.

Descriptive statistics are presented as means and standard deviations (SD), even if the intake does not, in all cases, fulfil the criterion of normal distribution but is slightly right skewed. The proportion of adolescents that adhered to recommended intake according to the 2023 Nordic Nutrition Recommendations (NNR) [24,25,26] for each micronutrient is presented.

Associations between selected dietary intake indicators and various wellbeing and health behaviour parameters were tested using chi-square tests in cross-tabulations. The selected dietary intake indicators were those macronutrients for which mean intake for both girls and boys was below the recommended intake plus fruit and vegetable consumption. For these analyses, we dichotomised daily dietary intake as follows: added sugars < 13% or ≥13% of total energy intake, saturated fat < 10% or ≥10%, omega 3 fatty acid intake ≥ 0.05% or <0.05%, dietary fibre ≥ 15 g/day or <15 g/day, eating fruit daily or not, eating vegetables daily or not, and fruit and vegetable intake ≥ 150 g/day or <150 g/day. The cut-points used for categorisation of dietary intake indicators were decided prior to conducting the analyses. This was carried out by dichotomising the variable as close to the recommended level as possible, while still obtaining comparison groups of a certain size. This was performed because, for some of the dietary indicators, no or extremely few participants complied with recommendations.

The following wellbeing and health behaviour parameters used for the analyses were also dichotomomised into yes vs. no: good self-rated health, headache, stomach ache, engages in sports, screen time < 4.5 h/day, packed lunch to school every day, buys food during school hours, ≥8 h of sleep on school nights.

Statistical analyses were made with the statistical program SSPS version 29.0, with statistical significance considered at *p* < 0.05.

## 3. Results

In this exploratory study, 81 students from four 8th-grade classes in the Faroe Islands participated. Three students did not submit the completed paper questionnaire containing information such as age and gender and thus were excluded. Out of the remaining 78 students, there were 33 boys (42%) and 45 girls (58%). They were, on average, 14.3 years old, 52 (67%) were 14 years old, and 26 (33%) were 15 years old.

The total daily energy intake for boys averaged 2224 kcal (lowest: 116.4, highest: 10,033.1, SD: 298.8), and girls’ daily energy intake averaged 1187 kcal (lowest 126.5, highest: 2859.0, SD: 99.2). While 24% of both girls and boys reported eating vegetables every day, 27% of girls and 30% of boys reported daily fruit consumption, and half of the participants reported not eating fruit or vegetables daily (56% of girls, 50% of boys). The anthropometric measurements showed that 11% of girls and 6% of boys were overweight or obese, according to the body mass index (BMI > 25), while 8% of girls and 18% of boys were underweight (BMI ≤ 18.5). 

Both boys and girls consumed more saturated fat on average than recommended, and girls’ intake of monounsaturated and polyunsaturated fatty acids was lower than recommended (Table 1). Both genders consumed too high amounts of sugar, too low amounts of omega-3 fatty acids, and too few dietary fibres compared to recommended levels.

Apart from phosphorus, girls’ intake of all vitamins and minerals was lower than the recommended intake, and for boys, this was the case for vitamins A, D, E, C, riboflavin, iodine, potassium, magnesium, and selenium (Table 2). The proportion of boys and girls that reported intake compliant with the recommendations was 9.1% for vitamin D. The corresponding proportions for girls and boys were for calcium 26.7% and 42.4%, for iron 2.2–11.1% and 44.4%, and for iodine 13.3% and 18.2%, respectively.

Our analyses of selected indicators of dietary intake in association with wellbeing and health behaviour parameters revealed statistically significant or borderline significant associations for saturated fat intake, dietary fibre intake, eating vegetables daily, and daily intake of fruits and vegetables (Table 3).

Regarding saturated fat intake, approximately one-third (36.1%) of those who had stomach aches kept their saturated fat intake below 10%, while this proportion was 76.5% for those without stomach aches. Of those who regularly engage in sports, just over half (54.5%) meet the recommendations for saturated fat intake, while this is the case for 31.3% of those who do not engage in sports. The proportion of pupils who rate their health as good and consume at least 15 g of dietary fibre is 40%, while it holds only for 13% of those who rate their health as less good. Additionally, half of those who do not have headaches consume the recommended amount of dietary fibre, while this is only the case for 26% of those who report having headaches. Out of those who rate their health as good, close to one-third consume vegetables daily (29%), while only 6.3% of those who rate their health as less good consume vegetables daily; almost half (47.1%) of those who report having stomach aches do not eat vegetables daily, while among those who eat vegetables daily, just under one-fifth (18%) experience stomach aches. The proportion of students who regularly engage in sports and also consume vegetables daily is almost one-third (31.8%), while out of those who do not engage in sports, only 12.5% consume vegetables daily. Of the students who sleep for a minimum of 8 h on weekdays, 37.9% also consume vegetables daily, while of those who sleep less than 8 h, only 17.4% consume vegetables daily. Sleep also relates to the intake of fruits and vegetables overall, in that 31% of those who sleep for at least 8 h on weekdays consume at least 150 g of fruits and vegetables daily, whereas those who sleep less than 8 h consume at least 150 g of fruits and vegetables in 10.9% of cases.

On the other hand, one-third (33.3%) of the pupils who bring packed lunches to school keep their saturated fat intake below the recommendations, while this applies to as many as 63.3% of those who do not.

When we stratified by gender (Supplementary Table S1), the differences in some associations appeared stronger for either boys or girls, whereas several associations were no longer significant.

## 4. Discussion

Even though this is a small and exploratory study, we report on the first data on the dietary intake of adolescents in the Faroe Islands, a small island nation in the Arctic. In this exploratory study, we implemented an online 24 h dietary recall tool in the Danish language. We obtained data on the dietary intake of 81 pupils from four 8th-grade classes in the Faroe Islands, representing their dietary intake on one weekday.

Furthermore, despite the small sample size, we observed associations between saturated fat intake, dietary fibre intake, and daily vegetable and fruit consumption with wellbeing and health behaviour parameters. For the most part, the associations were as we would expect: those with a healthier dietary intake were also more likely to report good self-rated health, to engage in sports, to sleep at least 8 h on school nights, and less likely to report headaches and stomach aches. Due to the cross-sectional study design, we are, however, unable to distinguish whether a healthier diet might be causing any effect on health parameters or vice versa. In addition, we are unable to take into account any underlying factors that may be associated with both, such as family socioeconomic status or physical activity.

Our findings indicate that most participating adolescents did not meet the NNR recommendations for intake of fruit and vegetables and most macro- and micronutrients. Despite being an exploratory study using one single 24 h dietary recall, our findings are in line with other larger and multinational studies, such as the Health Behaviour in School-aged Children (HBSC) studies conducted in collaboration with the WHO [28,29,30], including data from 11–15-year-olds from 45 countries, and the Healthy Lifestyle in Europe by Nutrition in Adolescence study including data from 12.5–17.5-year-olds from 10 large European cities [31,32,33]. There are few comparable studies from small isolated adolescent populations, but data from the Greenlandic HBSC studies indicate that the proportion of adolescents in Greenland never eating fruit was similar to the proportion of Faroese adolescents not eating fruit every day (56/50% of girls/boys in the Faroese data vs. 44% in 2006 in the Greenlandic data). When looking at daily fruit consumption, the proportion of Faroese adolescents reporting eating fruit every day was much higher among the Faroese adolescents compared to Greenlandic peers in the most recent survey (27/30% of girls/boys in the Faroese data vs. 18% in 2006 in the Greenlandic data) [34]. Interestingly, data from the surveys conducted on four occasions during the period of 1994–2006 in Greenland indicated a decrease in fruit consumption over the period, which we, unfortunately, cannot substantiate in the Faroes since there is no data available.

One main reason for targetting the data collection at 14–15-year-olds was that participants had to be able to use a relatively complex dietary recording form [35]. Additionally, recording intake in Danish might be a challenge to younger children. A study from 2016 described challenges for 11–12-year-olds in identifying certain types of foods, such as different types of milk and how food was prepared (fried, boiled, raw) [36]. On the other hand, a study among 11–16-year-olds showed that an online dietary registration form would be useful for this age group [37]. As identity development in adolescence is characterised by personal maturation and close and healthy family relationships and friendships are important for this development [38], we estimated that the chosen age group is appropriate with regard to preventive measures aimed at improving diet. This is further supported by data from the Greenlandic HBSC study from 2006, which showed that the prevalence of healthy dietary habits, such as low intake of sweets and sugar-sweetened beverages and eating fish at least once a week, decreased with increasing age [39]. In a 2018 report, the Danish Council for Health Promotion and Disease Prevention concluded that despite a positive development in dietary habits among Danish children and adolescents, only a few comply with the national dietary guidelines [17]. Although data are lacking for the Faroe Islands to depict any historical development, the present situation seems similar to neighbouring areas and calls for health promotion initiatives targeted at the younger population. To be effective, such initiatives should be complex. They should be targeted at multiple levels, including families/homes, schools/daycare, local communities, and at the societal level, and span from health education, using role models to enhancing access to healthy low-cost foods and meals, differentiated taxation of foods, statutory regulation ensuring smaller serving and pack sizes for unhealthy foods and beverages, and banning marketing of unhealthy foods and beverages targeted at adolescents, etc. [17].

### Strengths and Limitations

There are several strengths and limitations to our study. First, we conducted one single 24 h dietary recall, and secondly, the study sample is small, and thus limits us from drawing any firm conclusions. However, the participants constituted approx. 10% of those eligible for participation, as each year ~700 children are born in the Faroe Islands, and furthermore, schools from different parts of the islands were chosen with the aim of obtaining a representative sample. Further, we consider it a strength that we were able to detect statistically significant associations for several dietary indicators with health parameters despite the small sample size. With a significance level of 5%, we would expect 2–3 associations to be statistically significant due to mere chance, given the number of dietary indicators and health parameters in the analyses (Table 3). We observed ten statistically significant associations, which may imply that despite the small sample size, we did have sufficient statistical power for the analyses conducted.

Online dietary registration tools are constantly developing with the aim of rendering data as valid and as accurate as possible [40]. The online dietary recording tool used in this study, myfood24, analyses the intake of over 100 nutrients from the foods entered. The tool was developed around 2015 by researchers at the University of Leeds and has been validated in several populations and languages [20]. A study comparing the tool with interviewer-administered 24 h multiple-pass recall (*n* = 75, age 11–18 years) showed satisfactory agreement between the methods [36], and a study comparing the tool with weighed dietary records as well as biomarkers showed similar energy and macronutrient intake, and concluded that the validity of myfood24 is on par with other traditional dietary recording methods despite an underestimation for intake based on myfood24 for 15 different nutrients compared to the biomarkers [41]. One drawback with the tool is that dietary supplement use is not recorded, which may have caused misclassification of micronutrient intake for our participants, implying an underestimation of actual intake. Furthermore, total energy intake was relatively low, possibly due to underreporting, which might have been exacerbated as data collection was conducted in the classroom. In particular, for the girls, underreporting may partly explain their low intake of micronutrients, as their energy intake was relatively low in our data. Previous studies comparing data from different dietary assessment methods have indicated that several 24 h recalls, preferably representing both weekdays and weekends, are needed to obtain accurate data on habitual intake [21]. A statistical investigation of outliers only indicated one very high intake value in our data, which was not excluded from analyses since the participant had himself explained during the data collection that he had eaten unusually much during the previous day; one participant with a very low intake likewise told us that she had almost not eaten on the previous day. Therefore, all observations were kept for analyses despite considerable variability.

## 5. Conclusions

This exploratory study represents the first dietary assessment of adolescents in the Faroe Islands, addressing a significant gap in nutritional data for this population and laying the groundwork for future research in this field. Research investigating habitual dietary intake among adolescents is essential for planning targeted health promotion initiatives and preventive measures, especially given the shift from traditional seafood-based diets to a Western-style diet observed in adult populations in the area. Additionally, the novelty of this study is highlighted by the use of a web-based dietary assessment tool, which might potentially enhance data accuracy and accessibility but also demonstrate its feasibility for this age group. However, adjustments for the Faroese language, food culture, and traditional foods are necessary before applying this tool to larger samples of Faroese adolescent or even adult populations.

## Figures and Tables

**Table 1:** Macronutrient intake for 14–15-year-old participants in the study (*n* = 78) as mean and standard deviation (SD) percentage of total energy intake ^a^, and recommended intake according to the Nordic Nutrition Recommendations [24] for adults and children over 2 years [27] ^b^.

	Recommended Intake According to NNR	Girls (*n* = 45)	Boys (*n* = 33)
	% of Total Energy Intake or g/day	Mean % of Total Energy Intake or g/day (SD)	Mean % of Total Energy Intake or g/day (SD)
Total fat content	25–40%	31.4 (10.2)	28.6 (9.1)
Saturated fat	max 10%	**10.4 (4.8)** ^c^	**11.3 (4.9)** ^c^
Monounsaturated fat	10–20%	**9.2 (4.1)** ^c^	11.1 (4.9)
Polyunsaturated fat	5–10%	**3.7 (1.9)** ^c^	5.3 (2.6)
Omega 3	min 1%	**0.5 (0.5)** ^c^	**0.7 (0.7)** ^c^
Trans fatty acids	As low as possible	0.3 (0.4)	0.4 (0.7)
Carbohydrates	45–60%	55.1 (11.0)	**40.4 (11.4)** ^c^
Added sugar	max 10%	**18.4 (13.1)** ^c^	**17.0 (16.2)** ^c^
Dietary fibre (females) ^b^	25 g/day	**9.9 (6.6)** ^c^	n.a.
Dietary fibre (males) ^b^	35 g/day	n.a.	**19.4 (13.9)** ^c^
Protein	10–20%	14.5 (5.5)	16.2 (6.1)

^a^ Calculated based on mean total energy intake (kcal) in girls ($\overset{®}{x}$ = 1187) and boys ($\overset{®}{x}$ = 2224). ^b^ In most cases, the same recommendation applies to both genders, while there is differentiation between females and males in others. ^c^ Values that fall outside the recommendations are highlighted in bold.

**Table 2:** Micronutrient intake for 14–15-year-old participants in the study (*n* = 78) as mean and standard deviation (SD), recommended intake according to the Nordic Nutrition Recommendations [24,27] ^a,b^ and proportion of participants meeting the recommended intake.

	Recommended Amount/Day	Girls (*n* = 45)	Boys (*n* = 33)	Participants Meeting the Recommended Intake
**Vitamins, unit**		**Mean (SD)**	**Mean (SD)**	**%**
Vit. A, RE ^c^ (females) ^b^	700	**204.6 (234.5) ^d^**	n.a.	2.2
Vit. A, RE (males) ^b^	900	n.a.	**433.5 (826.1) ^d^**	11.1
Vit. D, µg	10	**0.7 (1.1) ^d^**	**2.6 (5.8) ^d^**	9.1
Vit. E, mg (females) ^b^	8	**2.9 (3.0) ^d^**	n.a.	4.4
Vit. E, mg (males) ^b^	10	n.a.	**6.9 (8.0) ^d^**	25.0
Vit. C, mg	75	**35.0 (61.0) ^d^**	**71.2 (122.9) ^d^**	19.8
Thiamin, mg (females) ^b^	1.1	**0.6 (0.4) ^d^**	n.a.	4.4
Thiamin, mg (males) ^b^	1.4	n.a.	1.4 (1.4)	25.0
Riboflavin, mg (females) ^b^	1.3	**0.8 (0.7) ^d^**	n.a.	13.3
Riboflavin, mg (males) ^b^	1.6	n.a.	**1.4 (1.5) ^d^**	22.2
Niacin, mg (females) ^b^	15	**11.3 (11.1) ^d^**	n.a.	13.3
Niacin, mg (males) ^b^	19	n.a.	19.8 (25.8)	27.3
Vit. B6, mg females) ^b^	1.2	**0.9 (1.9) ^d^**	n.a.	15.6
Vit. B6, mg (males) ^b^	1.5	n.a.	1.7 (1.8)	41.7
Folate fertile age, µg (females) ^b^	400	**93.3 (77.0) ^d^**	n.a.	0
B12, µg	2	**1.6 (1.9) ^d^**	4.9 (10.1)	41.3
**Minerals, unit**				
Calcium, mg	800	**581.5 (438.8) ^d^**	851.7 (651.6)	33.3
Phosphorus, mg	600	710.7 (452.9)	1265.6 (965.0)	65.4
Iron, mg (females) ^b^	9–15	**4.67 (3.2)** ^d^	n.a.	≥9 mg: 11.1 ≥15 mg ^e^: 2.2
Iron, mg (males) ^b^	9	n.a.	9.0 (7.0)	44.4
Iodine, µg	150	**74.6 (76.3)** ^d^	**109.3 (94.3)** ^d^	15.3
Potassium, g (females) ^b^	3.1	**1.3 (0.9)** ^d^	n.a.	27.3
Potassium, g (males) ^b^	3.5	n.a.	**2.6 (1.8)** ^d^	15.2
Magnesium, mg (females) ^b^	280	**148.0 (90.0)** ^d^	n.a.	11.1
Magnesium, mg (males) ^b^	350	n.a.	**272.0 (185.3)** ^d^	19.4
Selenium, µg (females) ^b^	50	**17.7 (13.1)** ^d^	n.a.	0
Selenium, µg (males) ^b^	60	n.a.	**43.5 (48.6)** ^d^	16.7
Zink, mg (females) ^b^	7	**5.2 (3.7)** ^d^	n.a.	24.4
Zink, mg (males) ^b^	9	n.a.	10.0 (8.6)	38.9

^a^ No nutritional recommendations were found specifically for the age group in the study. ^b^ In some cases, the same recommendation applies to both genders, while in others, there is differentiation between females and males. ^c^ Vitamin A intakes or requirements are generally expressed in terms of retinol equivalents (RE). One RE is defined as the biological activity associated with 1 µg of all-trans retinol. ^d^ Values that fall outside the recommendations are highlighted in bold. ^e^ For women, the recommendation is 15 mg/day after menarche.

**Table 3:** The percentage of participants (*n* = 78) fulfilling selected aspects of dietary intake (added sugar max 13%, saturated fat max 10%, omega-3 fatty acids min 0.5% of total energy intake (OTEI), dietary fibre min 15 g/day, eat fruits and vegetables every day, fruit and vegetable consumption min 150 g/day) in association with wellbeing and health behaviour parameters.

	Added Sugar max. 13% OTEI ^a^ (rec: max 10%) ^b^	*p*	Saturated Fat max 10% OTEI	*p*	Omega-3 min. 0.5% OTEI (rec: min 1%)	*p*	Dietary Fibre min. 15 g (rec: 30 g/day)	*p*	Eats Fruit Every Day	*p*	Eats Vegetables Every Day	*p*	Fruit and Vegetables min. 150 g/day (rec. 600 g/day)	*p*
	**%**		**%**		**%**		**%**		**%**		**%**		**%**	
**Good self-rated health**														
No	37.5		37.5		50.0		**12.5**		31.3		**6.3**		6.3	
Yes	33.9	n.s.	46.8	n.s.	40.3	n.s.	**40.3**	**0.037**	27.4	n.s.	**29.0**	**0.058 ^d^**	21.0	n.s.
**Headache**														
No	32.1		46.4		42.9		**50.0**		28.6		17.9		14.3	
Yes	36.0	n.s.	44.0	n.s.	42.0	n.s.	**26.0**	**0.033**	28.0	n.s.	28.0	n.s.	20.0	n.s.
**Stomach ache**														
No	35.3		**76.5 ^c^**		41.2		41.2		23.5		**47.1**		17.6	
Yes	34.4	n.s.	**36.1**	**0.003**	42.6	n.s.	32.8	n.s.	29.5	n.s.	**18.0**	**0.014**	18.0	n.s.
**Engage in sports**														
No	40.6		**31.3**		46.9		28.1		18.8		**12.5**		9.4	
Yes	27.3	n.s.	**54.5**	**0.044**	36.4	n.s.	38.6	n.s.	36.4	n.s.	**31.8**	**0.05**	25.0	n.s.
**Screen time ≤ 4.5 h/day**														
No	31.4		37.1		40.0		25.7		20.0		20.0		8.6	
Yes	34.2	n.s.	52.6	n.s.	36.8	n.s.	42.1	n.s.	36.8	n.s.	28.9	n.s.	23.7	n.s.
**Packed lunch daily**														
No	40.0		**63.3**		43.3		23.3		20.0		16.7		16.7	
Yes	31.3	n.s.	**33.3**	**0.01**	41.7	n.s.	41.7	n.s.	33.3	n.s.	29.2	n.s.	18.8	n.s.
**Buys food during school hours**														
No	32.6		51.2		44.2		39.5		32.6		25.6		18.6	
Yes	38.2	n.s.	38.2	n.s.	41.2	n.s.	26.5	n.s.	23.5	n.s.	23.5	n.s.	17.6	n.s.
**Sleep ≥ 8 h on weeknights**														
No	39.1		45.7		43.5		32.6		23.9		**17.4**		**10.9**	
Yes	27.6	n.s.	41.4	n.s.	37.9	n.s.	41.4	n.s.	37.9	n.s.	**37.9**	**0.046**	**31.0**	**0.029**

^a^ OTEI: of total energy intake. ^b^ The minimum cut-off value does not always correspond to the recommendations, as the numbers were too small. ^c^ The *p*-value is indicated to the right of the result and highlighted in bold in case of statistical significance observed in Pearson chi-square, Asymptotic Significance (2-sided); when not statistically significant n.s. is stated. ^d^ Borderline statistically significant.

## Data Availability

The data presented in this study are available on request from the corresponding author due to legal restrictions and ethical concerns.

## Supplementary Materials

The following supporting information can be downloaded at: https://www.mdpi.com/article/10.3390/nu16162621/s1, Table S1: The percentage of participants (*n* = 78, 33 boys, 45 girls) fulfilling selected aspects of dietary intake in association with health behaviour parameters stratified by gender (statistically significant associations highlighted). References [26,27] are cited in Supplementary Materials.
