# Supplementary material for: Dietary Intake of 14–15-Year-Old Faroese Adolescents by an Online Assessment Tool and Associations with Wellbeing and Health Behaviour
Source: Nutrients. 2024 Aug 9;16(16):2621. doi: 10.3390/nu16162621 (PMC11357399; doi:10.3390/nu16162621)
Supplement: Supplementary file 1 [file nutrients-16-02621-s001.zip › nutrients-3036916-supplementary.pdf]

Supplementary Table S1: The percentage of participants (n=78, 33 boys, 45 girls) fulfilling selected aspects of dietary intake in association with health behaviour parameters stratified by gender (statistically significant associations highlighted).

	Saturated fat max 10% OTEI[a]	Dietary fibre min. 15 g (rec: 30g/day)	Eats vegetables everyday	Fruit & vegetables min. 150 g/day (rec.600g/day)
	Boys/Girls %	Boys/Girls %	Boys/Girls %	Boys/Girls %
Good selfrated health				
No		**0.0**[d]/15.4	0.0/7.7	
Yes		**66.7**/15.6	26.7/31.3	
Headache				
No		57.9/33.3		
Yes		64.3/11.1		
Stomach ache				
No	**81.8**[b]/66.7		**45.5**[e]/50.0	
Yes	**27.3**/41		**13.6**/20.5	
Engage in sports				
No	27.3/33.3		18.2/**9.5**[f]	
Yes	52.4/56.5		23.8/**39.1**	
Packed lunch daily				
No	60./**65.0**[c]			
Yes	45.4/**28.0**			
Sleep ≥ 8 h on weeknights				
No				**11.8**[g]/10.3
Yes				**46.7**/14.3

[a] OTEI: Of Total Energy Intake.

[b]P-value=0,003 [c]P-value=0,013 [d]P-value=0,024 [e]P-value=0,044 [f]P-value=0,023 [g]P-value=0,028

1. Nordiske Næringsstofsanbefalinger. 2023 (Nordic Nutritional Recommendations 2023). Available online: https://www.sundhed.dk/sundhedsfaglig/laegehaandbogen/sundhedsoplysning/sundhedsoplysning/kost/nordiske-naeringsstofsanbefalinger/ (accessed on 21 July 2024).

2. Sundhedsstyrelsen (the Danish Health Authority). Anbefalinger om Kost—De Officielle Kostråd. 2021. Available online: https://sst.dk/da/Borger/En-sund-hverdag/Kost/Anbefalinger-om-kost (accessed on 21 July 2024). Available online in English from the Danish Veterinay and Food Administration https://en.foedevarestyrelsen.dk/food/nutrition-and-health/the-official-dietary-guidelines
